# Supplementary material for: Integrating niche and occupancy models to infer the distribution of an endemic fossorial snake (Atractus lasallei)
Source: PLoS One. 2024 Aug 20;19(8):e0308931. doi: 10.1371/journal.pone.0308931 (PMC11335104; doi:10.1371/journal.pone.0308931)
Supplement: S1 Table — (DOCX) [file pone.0308931.s001.docx]

**S1.** **Environmental variables for niche and occupancy models**

| **Variables** | **Code** | **Occupancy models** | | **Niche models** | **Source** |
| --- | --- | --- | --- | --- | --- |
|  |  | **Occupancy Sub-models** | **Detectability Sub-models** |  |  |
| Terrain slope | Slope | X |  | X | (Amatulli et al., 2020) |
| Topographic convergence | Con | X |  | X | (Amatulli et al., 2020) |
| Compound Topographic Index | CTI | X |  | X | (Amatulli et al., 2020) |
| Minimum temperature of the coldest month (ground_level) | Tmin | X |  |  | (Lembrechts et al., 2021)^1^ |
| Annual mean soil temperature | Tprom | X |  |  | (Lembrechts et al., 2021) ^1^ |
| Maximum temperature of the warmest month (ground-level) | Tmax | X |  | X (SET 1) | (Lembrechts et al., 2021) ^1^ |
| Temperature seasonality (ground-level) |  |  |  | X (SET 1) | (Lembrechts et al., 2021) ^1^ |
| Maximum temperature |  |  |  | X (SET 2) | (Karger et al., 2021b)^2^ |
| Temperature seasonality |  |  |  | X (SET 2) | (Karger et al., 2021b) ^2^ |
| Temperature (ground) | T_ground |  | X |  | HOBO proV2 |
| Soil moisture | Soil_moisture |  | X |  | HOBO proV2 |
| Distance to nearest bodies of water (m) | D_water | X |  |  | QGIS |
| Distance to the nearest house (m) | D_house | X |  |  | QGIS |
| Distance to nearest forest (m) | D_forest | X |  |  | QGIS |
| Vegetation height (m) | Veg_H | X | X |  | (Potapov et al., 2020) |
| Leaf litter depth (cm) | Leaf_Dep | X |  |  | Barreno |
| Depth of the zero horizon (cm) | Hori0 | X |  |  | Barreno |
| Average annual precipitation (mm) |  |  |  | X | (Karger et al., 2021a) ^2^ |
| Evapotranspiration (mm per month) |  |  |  | X | (Karger et al., 2021a) ^2^ |
| Organic carbon in soil |  |  |  | X | (Poggio et al., 2021) |
| Number of cover objects | N_obj |  | X |  | Field |

^1^ Ground-level Temperature data were taken from 2000-2020 (Lembrechts et al. 2021).

^2^ Atmospheric Temperature, annual precipitation, and evapotranspiration (Karger et al. 2021a) refer to long term averages (1980-2010).
