# Supplementary material for: Integrating niche and occupancy models to infer the distribution of an endemic fossorial snake (Atractus lasallei)
Source: PLoS One. 2024 Aug 20;19(8):e0308931. doi: 10.1371/journal.pone.0308931 (PMC11335104; doi:10.1371/journal.pone.0308931)
Supplement: S2 Table — (DOCX) [file pone.0308931.s002.docx]

**S4: Biologically plausible and simple models**

Models tested in the occupancy modeling process.

| **Model** | **Detection** | **Occupancy** |
| --- | --- | --- |
| 1 | ~1 | ~1 |
| 2 | ~T_ground | ~1 |
| 3 | ~Soil_moisture | ~1 |
| 4 | ~N_obj | ~1 |
| 5 | ~Veg_H | ~1 |
| 6 | ~Veg_H_buffer50m | ~1 |
| 7 | ~Veg_H_buffer100m | ~1 |
| 8 | ~Veg_H_buffer200m | ~1 |
| 9 | ~Veg_H_buffer300m | ~1 |
| 10 | ~Veg_H_buffer400m | ~1 |
| 11 | ~Veg_H_buffer500m | ~1 |
| 12 | ~Veg_H_buffer1000m | ~1 |
| 13 | ~Veg_H+N_obj | ~1 |
| 14 | ~Veg_H_buffer50m+N_obj | ~1 |
| 15 | ~Veg_H_buffer100m+N_obj | ~1 |
| 16 | ~Veg_H_buffer200m+N_obj | ~1 |
| 17 | ~Veg_H_buffer300m+N_obj | ~1 |
| 18 | ~Veg_H_buffer400m+N_obj | ~1 |
| 19 | ~Veg_H_buffer500m+N_obj | ~1 |
| 20 | ~Veg_H_buffer1000m+N_obj | ~1 |
| 21 | ~N_obj | ~Slope |
| 22 | ~N_obj | ~Con |
| 23 | ~N_obj | ~ITC |
| 24 | ~N_obj | ~Tmin |
| 25 | ~N_obj | ~Tprom |
| 26 | ~N_obj | ~Tmax |
| 27 | ~N_obj | ~D_water |
| 28 | ~N_obj | ~D_house |
| 29 | ~N_obj | ~D_forest |
| 30 | ~N_obj | ~Leaf_Dep |
| 31 | ~N_obj | ~Hori0 |
| 32 | ~N_obj | ~Veg_H |
| 33 | ~N_obj | ~Veg_H_buffer50m |
| 34 | ~N_obj | ~Veg_H_buffer100m |
| 35 | ~N_obj | ~Veg_H_buffer200m |
| 36 | ~N_obj | ~Veg_H_buffer300m |
| 37 | ~N_obj | ~Veg_H_buffer400m |
| 38 | ~N_obj | ~Veg_H_buffer500m |
| 39 | ~N_obj | ~Veg_H_buffer1000m |
| 40 | ~N_obj | ~Veg_H+Slope |
| 41 | ~N_obj | ~Veg_H+Con |
| 42 | ~N_obj | ~Veg_H+CTI |
| 43 | ~N_obj | ~Veg_H+Tmin |
| 44 | ~N_obj | ~Veg_H+Tprom |
| 45 | ~N_obj | ~Veg_H+Tmax |
| 46 | ~N_obj | ~Veg_H+D_water |
| 47 | ~N_obj | ~Veg_H+D_house |
| 48 | ~N_obj | ~Veg_H+D_forest |
| 49 | ~N_obj | ~Veg_H+Leaf_Dep |
| 50 | ~N_obj | ~Veg_H+Hori0 |
| 51 | ~N_obj | ~Veg_H_buffer50m+Slope |
| 52 | ~N_obj | ~Veg_H_buffer50m+Con |
| 53 | ~N_obj | ~Veg_H_buffer50m+CTI |
| 54 | ~N_obj | ~Veg_H_buffer50m+Tmin |
| 55 | ~N_obj | ~Veg_H_buffer50m+Tprom |
| 56 | ~N_obj | ~Veg_H_buffer50m+Tmax |
| 57 | ~N_obj | ~Veg_H_buffer50m+D_water |
| 58 | ~N_obj | ~Veg_H_buffer50m+D_house |
| 59 | ~N_obj | ~Veg_H_buffer50m+D_forest |
| 60 | ~N_obj | ~Veg_H_buffer50m+Leaf_Dep |
| 61 | ~N_obj | ~Veg_H_buffer50m+Hori0 |
| 62 | ~N_obj | ~CTI+Tprom |
| 63 | ~N_obj | ~CTI+D_forest |
| 64 | ~N_obj | ~CTI+Leaf_Dep |
| 65 | ~N_obj | ~Tprom+D_forest |
| 66 | ~N_obj | ~Tprom+Leaf_Dep |
| 67 | ~N_obj | ~D_forest+Leaf_Dep |
| 68 | ~N_obj | ~Leaf_Dep+Slope |
| 69 | ~N_obj | ~Leaf_Dep+Con |
| 70 | ~N_obj | ~Leaf_Dep+CTI |
| 71 | ~N_obj | ~Leaf_Dep+Tmin |
| 72 | ~N_obj | ~Leaf_Dep+Tprom |
| 73 | ~N_obj | ~Leaf_Dep+Tmax |
| 74 | ~N_obj | ~Leaf_Dep+D_water |
| 75 | ~N_obj | ~Leaf_Dep+D_house |
| 76 | ~N_obj | ~Leaf_Dep+D_forest |
| 77 | ~N_obj | ~Veg_H+Slope |
| 78 | ~N_obj | ~Veg_H+Con |
| 79 | ~N_obj | ~Veg_H+CTI |
| 80 | ~N_obj | ~Veg_H+Tmin |
| 81 | ~N_obj | ~Veg_H+Tprom |
| 82 | ~N_obj | ~Veg_H+Tmax |
| 83 | ~N_obj | ~Veg_H+D_water |
| 84 | ~N_obj | ~Veg_H+D_house |
| 85 | ~N_obj | ~Veg_H+D_forest |
| 86 | ~N_obj | ~Veg_H+Leaf_Dep |
| 87 | ~N_obj | ~Veg_H+Hori0 |
| 88 | ~N_obj | Tprom+Hori0+Slope+D_house  +CTI+D_forest |
