# Supplementary material for: Integrating niche and occupancy models to infer the distribution of an endemic fossorial snake (Atractus lasallei)
Source: PLoS One. 2024 Aug 20;19(8):e0308931. doi: 10.1371/journal.pone.0308931 (PMC11335104; doi:10.1371/journal.pone.0308931)
Supplement: S3 Table — (DOCX) [file pone.0308931.s003.docx]

**S5:** *Atractus lasallei* voucher and iNaturalist observations used for niche modelling

Reviewed records from biological collections and iNaturalist. Data doi: https://doi.org/10.15468/dl.jxppaq

| **Reviewed specimens of A. lasallei** | **Year** |
| --- | --- |
| http://www.inaturalist.org/observations/3740321 | 2016 |
| https://www.inaturalist.org/observations/102839645 | 2021 |
| https://www.inaturalist.org/observations/108206985 | 2022 |
| https://www.inaturalist.org/observations/18712973 | 2018 |
| https://www.inaturalist.org/observations/22172079 | 2019 |
| https://www.inaturalist.org/observations/22301170 | 2019 |
| https://www.inaturalist.org/observations/23145305 | 2019 |
| https://www.inaturalist.org/observations/23612922 | 2019 |
| https://www.inaturalist.org/observations/25183833 | 2019 |
| https://www.inaturalist.org/observations/36348831 | 2019 |
| https://www.inaturalist.org/observations/36704394 | 2019 |
| https://www.inaturalist.org/observations/38030501 | 2020 |
| https://www.inaturalist.org/observations/39481854 | 2020 |
| https://www.inaturalist.org/observations/41802482 | 2020 |
| https://www.inaturalist.org/observations/45871052 | 2020 |
| https://www.inaturalist.org/observations/66493556 | 2020 |
| https://www.inaturalist.org/observations/67010601 | 2020 |
| https://www.inaturalist.org/observations/67010603 | 2020 |
| https://www.inaturalist.org/observations/69304474 | 2021 |
| https://www.inaturalist.org/observations/69883388 | 2021 |
| https://www.inaturalist.org/observations/79426837 | 2021 |
| https://www.inaturalist.org/observations/8434656 | 2017 |
| https://www.inaturalist.org/observations/8820108 | 2017 |
| https://www.inaturalist.org/observations/90425085 | 2021 |
| https://www.inaturalist.org/observations/92367444 | 2021 |
| ICN10618 | 1981 |
| ICN10620 | 1981 |
| ICN10629 | 1985 |
| ICN10713 | 1981 |
| ICN10714 | 1981 |
| ICN5674 | 1980 |
| CBUCES-D-272 | NA |
| CBUCES-D-273 | NA |
| CBUCES-D-No ingresado | NA |
| CBUCES-D-No ingresado | NA |
| CBUCES-D-No ingresado (a001) | NA |
| CBUCES-D-No ingresado(a003) | NA |
| LaSalle:MLS-ofi:135 | 1924 |
| LaSalle:MLS-ofi:1765 | 1963 |
| LaSalle:MLS-ofi:1781 | 1963 |
| LaSalle:MLS-ofi:1782 | 1963 |
| LaSalle:MLS-ofi:1783 | 1963 |
| LaSalle:MLS-ofi:1842 | 1962 |
| LaSalle:MLS-ofi:1843 | 1963 |
| LaSalle:MLS-ofi:1856 | 1963 |
| LaSalle:MLS-ofi:1878 | 1965 |
| LaSalle:MLS-ofi:1902 | 1965 |
| LaSalle:MLS-ofi:1903 | 1965 |
| LaSalle:MLS-ofi:1904 | 1965 |
| LaSalle:MLS-ofi:1941 | 1965 |
| LaSalle:MLS-ofi:1946 | 1966 |
| LaSalle:MLS-ofi:202 | 1937 |
| LaSalle:MLS-ofi:203 | 1938 |
| LaSalle:MLS-ofi:204 | 1937 |
| LaSalle:MLS-ofi:205 | 1938 |
| LaSalle:MLS-ofi:206 | 1939 |
| LaSalle:MLS-ofi:207 | 1935 |
| LaSalle:MLS-ofi:2077 | NA |
| LaSalle:MLS-ofi:208 | 1938 |
| LaSalle:MLS-ofi:209 | 1938 |
| LaSalle:MLS-ofi:2129 | 1971 |
| LaSalle:MLS-ofi:219 | 1938 |
| LaSalle:MLS-ofi:2210 | NA |
| LaSalle:MLS-ofi:2217 | 1972 |
| LaSalle:MLS-ofi:223 | 1937 |
| LaSalle:MLS-ofi:230 | NA |
| LaSalle:MLS-ofi:2356 | 1936 |
| LaSalle:MLS-ofi:2412 | 1981 |
| LaSalle:MLS-ofi:280 | 1938 |
| LaSalle:MLS-ofi:2829 | 1963 |
| LaSalle:MLS-ofi:2939 | 1954 |
| LaSalle:MLS-ofi:2941 | 1963 |
| LaSalle:MLS-ofi:2944 | 1968 |
| LaSalle:MLS-ofi:2946 | 1970 |
| LaSalle:MLS-ofi:2955 | 1981 |
| LaSalle:MLS-ofi:2958 | 1982 |
| LaSalle:MLS-ofi:3017 | NA |
| LaSalle:MLS-ofi:303 | 1935 |
| MCZ:Herp:R-32799 | 1919-1974 |
| MHUA-R 14003 | 1997 |
| MHUA-R 14028 | 1997 |
| MHUA-R 14060 | 1998 |
| MHUA-R 14086 | 1999 |
| MHUA-R 14112 | 2001 |
| MHUA-R 14194 | 2001 |
| MHUA-R 14221 | 2002 |
| MHUA-R 14299 | 2004 |
| MHUA-R 14368 | 2005 |
| MHUA-R 14383 | 2005 |
| MHUA-R 14542 | 2007 |
| MHUA-R 14550 | NA |
| MHUA-R 14854 | 2011 |
| MHUA-R 14895 | 2012 |
| MHUA-R 14959 | 2014 |
| MHUA-R 15145 | 2016 |
| MHUA-R 15499 | 2019 |
| MHUA-R 15500 | 2019 |
| CSJ-h 3966 | 1930 |
| CSJ-h 4037 | 1932 |
| CSJ-h 4091 | 1995 |
| CSJ-h 4173 | 1986 |
| CSJ-h 4250 | 1978 |
| CSJ-h 4346 | 1998 |
| CSJ-h 4347 | NA |
| CSJ-h 4350 | 1998 |
| CSJ-h 4536 | 1972 |
| CSJ-h 4663 | 2000 |
| CSJ-h 4671 | 1998 |
