# Supplementary material for: Integrating niche and occupancy models to infer the distribution of an endemic fossorial snake (Atractus lasallei)
Source: PLoS One. 2024 Aug 20;19(8):e0308931. doi: 10.1371/journal.pone.0308931 (PMC11335104; doi:10.1371/journal.pone.0308931)
Supplement: S4 Table — (DOCX) [file pone.0308931.s004.docx]

**S9: Scale count from specimens obtained in the Western Andes.**

General lepidosis of specimens from biological collections whose locality was in the Western Cordillera.

| **ID** | **Locality** | **Dorsal scales** | **Ventral scales** | **Caudal scales** | **Preventral scales** | **Supralabial scales** | **Infralabial scales** |
| --- | --- | --- | --- | --- | --- | --- | --- |
| MHUA-R 15499 | Peque | 17 | 162 | 24 |  | 7 | 6 |
| MHUA-R 15500 | Peque | 17 | 168 | 24 |  | 7 | 6 |
| MLS-ofi:303 | Jericó | 17 | 166 | 15 | 3 | 7 | 6 |
| CSJ-h 3966 | Jericó | 17 | 168 | 17 | 3 | 7 | 7 |
| CSJ-h 4037 | Jericó | 17 | 164 | 23 | 4 | 7 | 7 |
| CSJ-h 4347 | Jericó | 17 | 168 | 15 | 4 | 7 | 7 |
