# Supplementary material for: Integrating niche and occupancy models to infer the distribution of an endemic fossorial snake (Atractus lasallei)
Source: PLoS One. 2024 Aug 20;19(8):e0308931. doi: 10.1371/journal.pone.0308931 (PMC11335104; doi:10.1371/journal.pone.0308931)
Supplement: S1 File — (DOCX) [file pone.0308931.s005.docx]

**S2: Spearman correlation test for niche modeling variable selection**


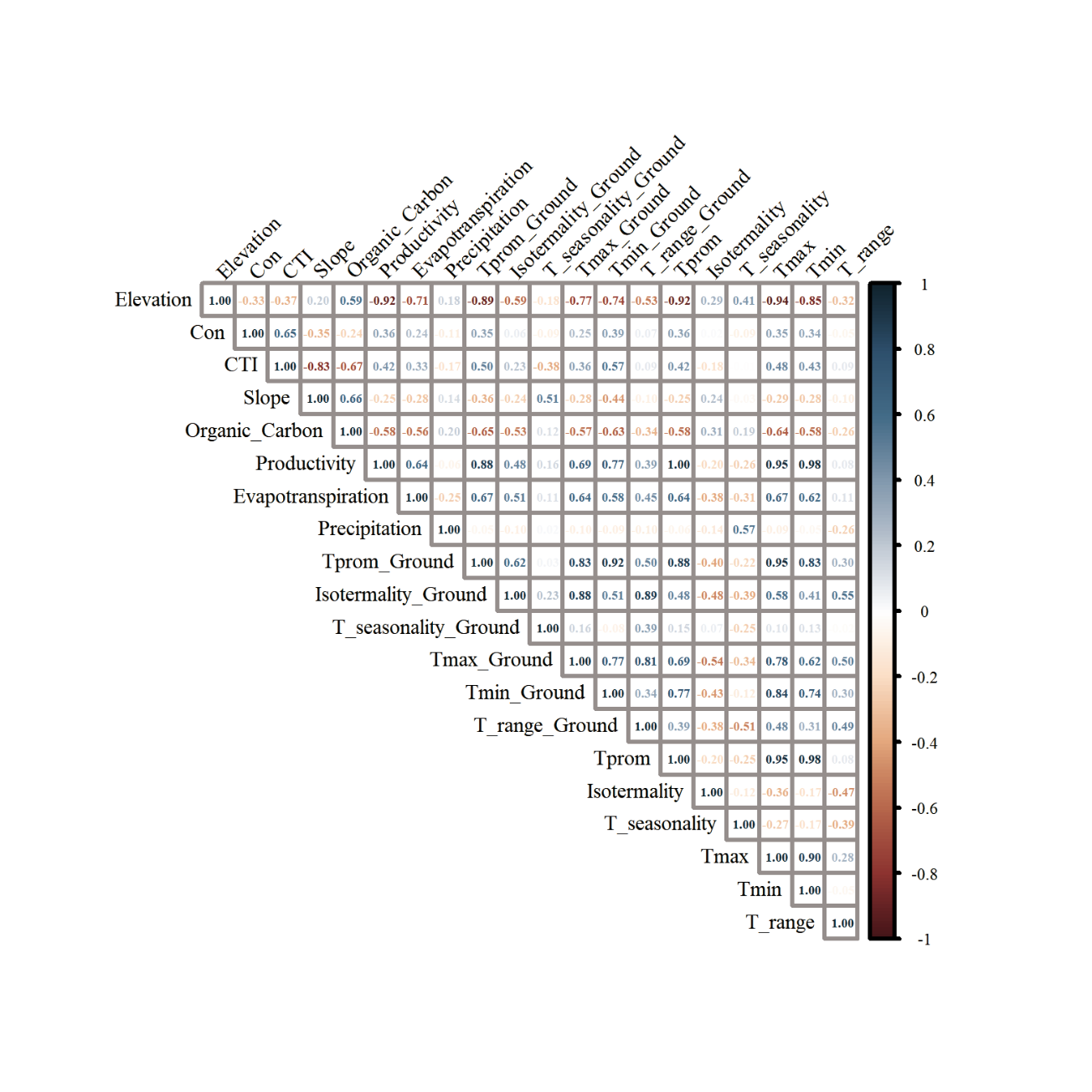


**Fig. S1.** Spearman correlation matrix between all pairs of variables considered in the construction of potential distribution models. Variables with a collinearity value greater than 0.8 or less than -0.8 were considered correlated.


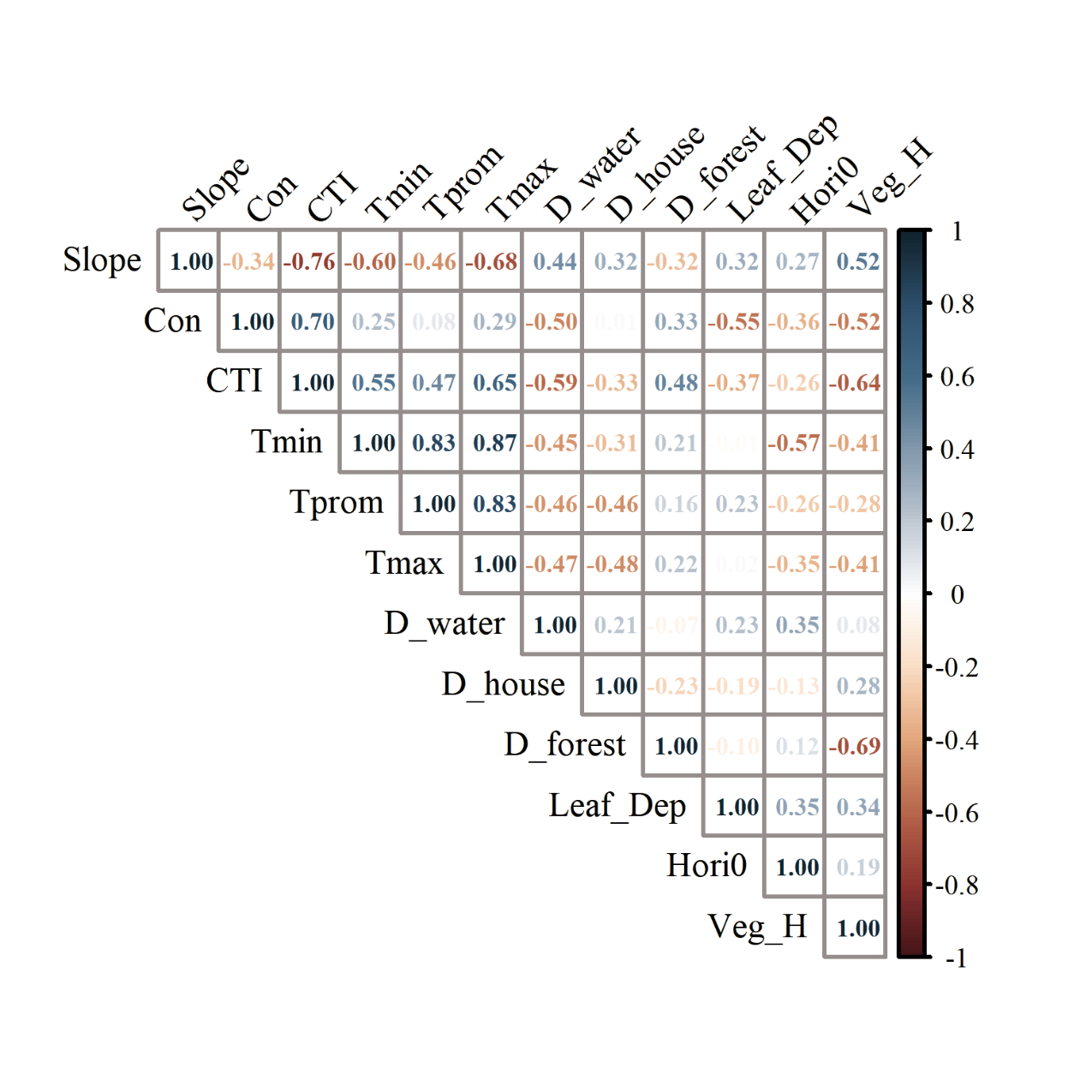


**Fig. S2.** Spearman correlation matrix between all pairs of variables considered in the construction of sub-models of occupancy. Variables with a collinearity value greater than 0.8 or less than -0.8 were considered correlated.
