# Supplementary material for: Integrating niche and occupancy models to infer the distribution of an endemic fossorial snake (Atractus lasallei)
Source: PLoS One. 2024 Aug 20;19(8):e0308931. doi: 10.1371/journal.pone.0308931 (PMC11335104; doi:10.1371/journal.pone.0308931)
Supplement: S1 Fig — (DOCX) [file pone.0308931.s006.docx]

**S3: Artificial cover objects during the sample phase for occupancy models**


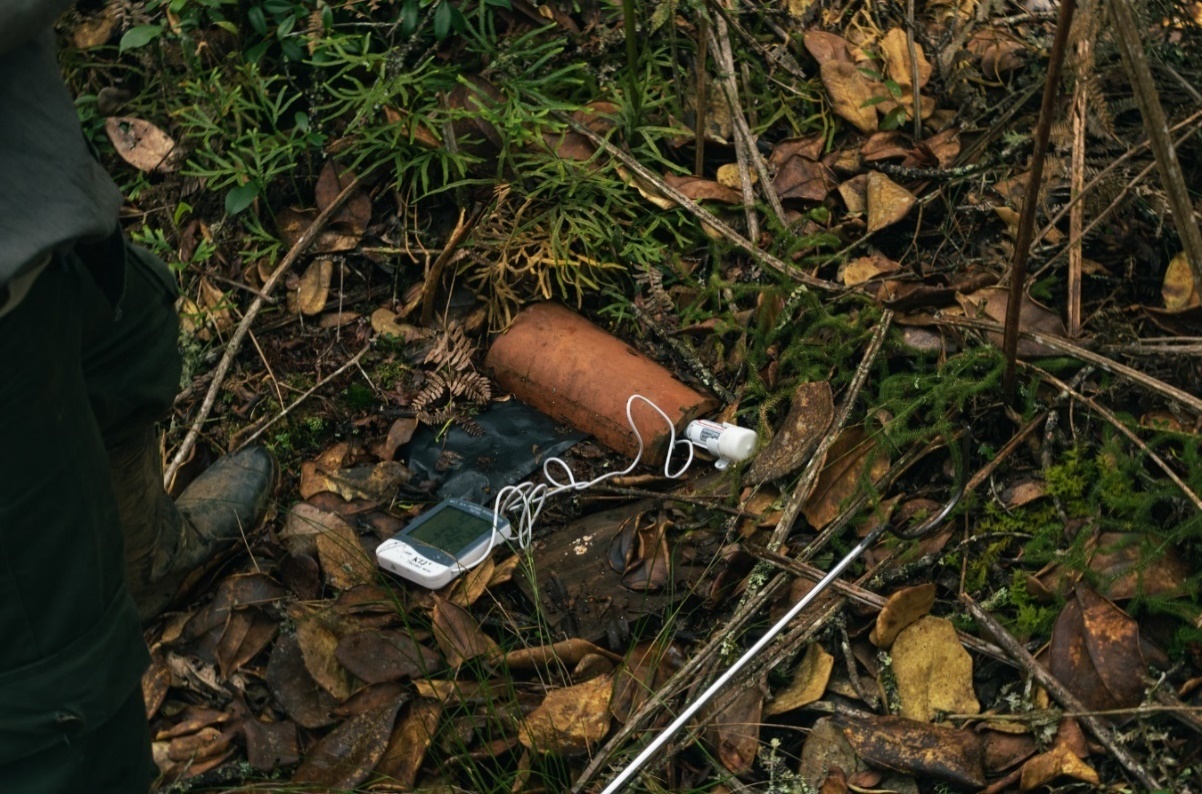


**Fig. S3**. Artificial cover objects used to increase the snake's detectability (roof tile, board, and plastic sheet).
