# Supplementary material for: Integrating niche and occupancy models to infer the distribution of an endemic fossorial snake (Atractus lasallei)
Source: PLoS One. 2024 Aug 20;19(8):e0308931. doi: 10.1371/journal.pone.0308931 (PMC11335104; doi:10.1371/journal.pone.0308931)
Supplement: S2 Fig — (DOCX) [file pone.0308931.s007.docx]

**S6: Response curves of the most important variables from selected niche models**


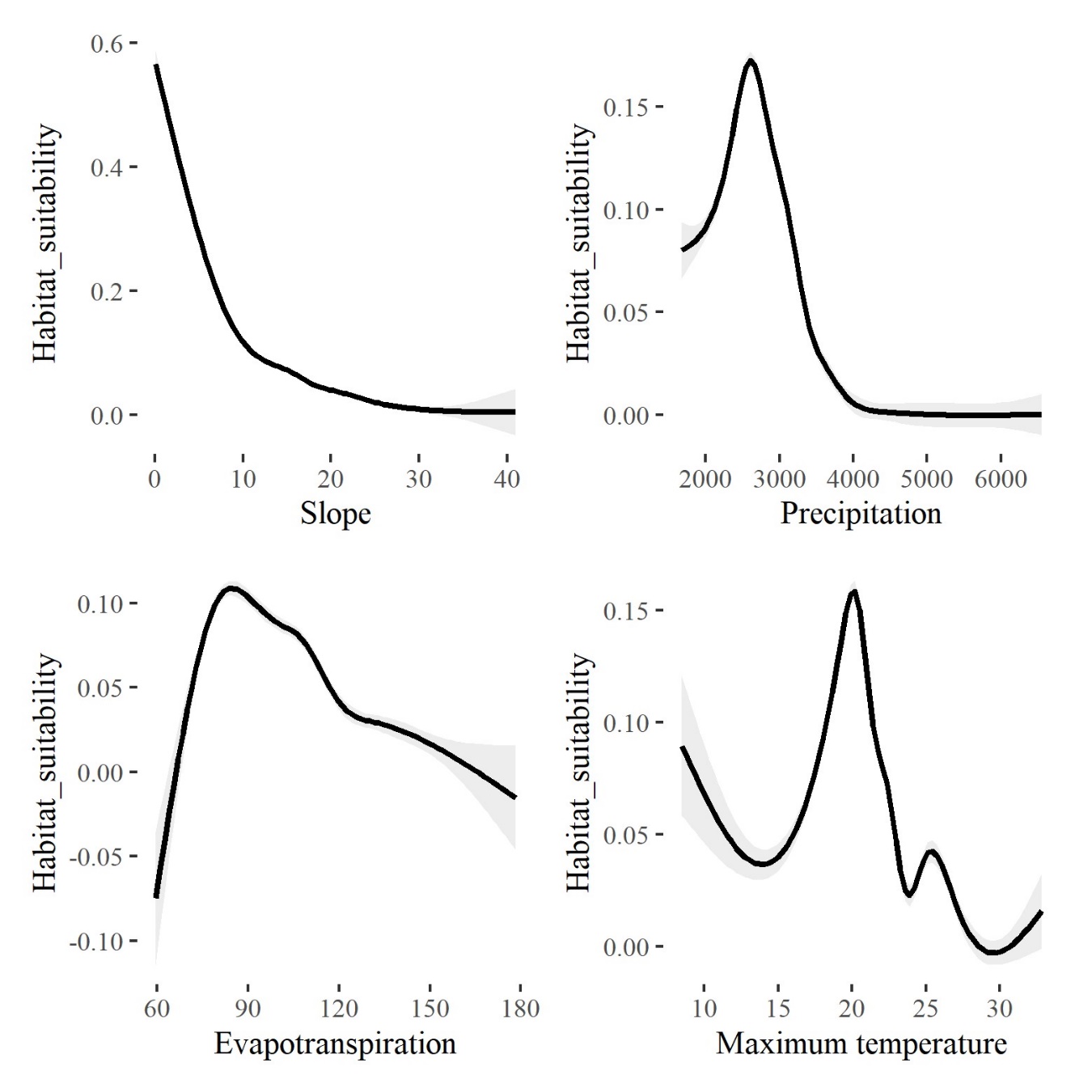


**Fig. S5.** Response curves of the four variables with the highest gain for the best niche model. The gray shadow represents the variation in the response curves among ten replicates.
