# Supplementary material for: Integrating niche and occupancy models to infer the distribution of an endemic fossorial snake (Atractus lasallei)
Source: PLoS One. 2024 Aug 20;19(8):e0308931. doi: 10.1371/journal.pone.0308931 (PMC11335104; doi:10.1371/journal.pone.0308931)
Supplement: S3 Fig — (DOCX) [file pone.0308931.s008.docx]

**S7: Detection history from occupancy model sampling phase.**


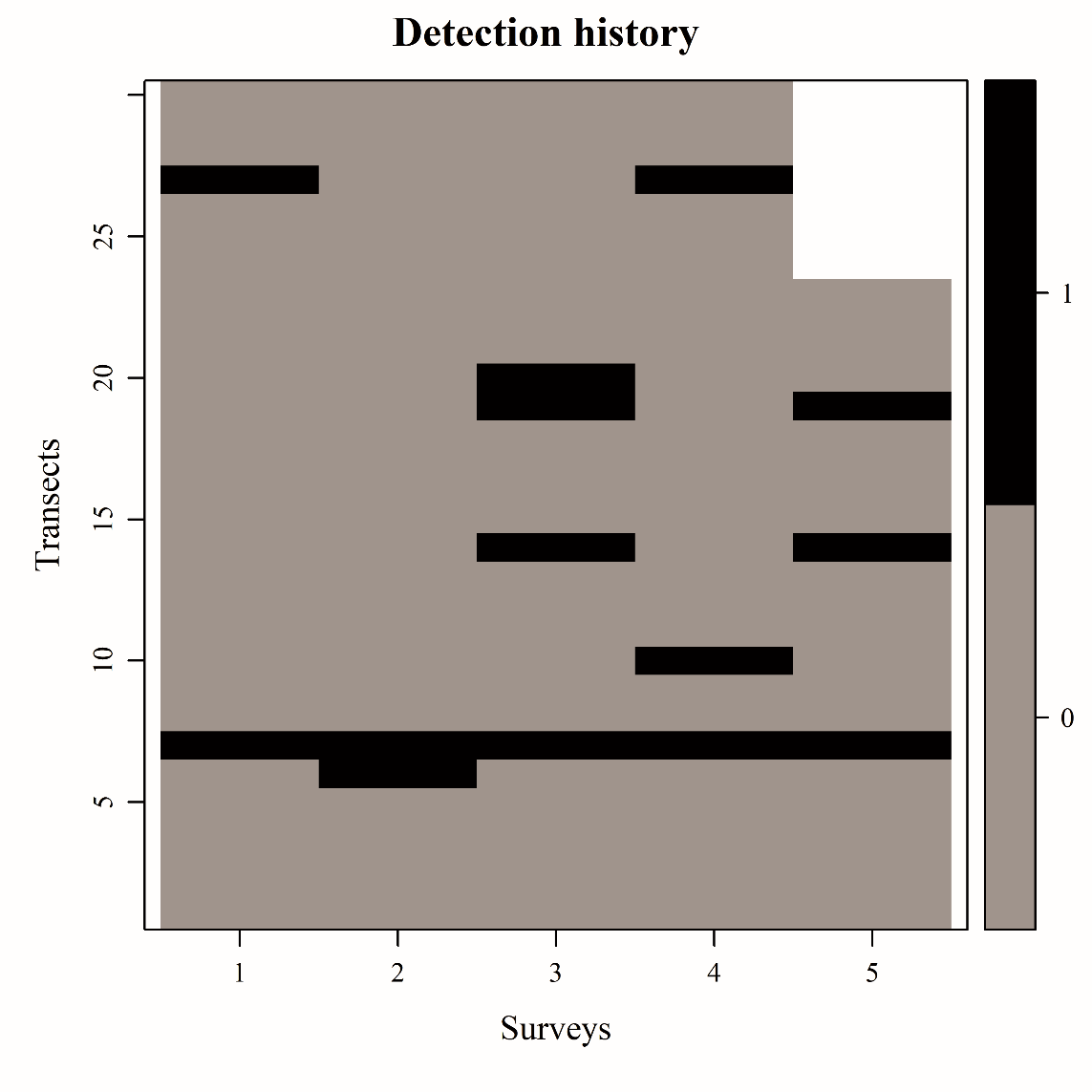


**Fig. S6.** Detection history from the occupancy model sampling scheme in San Pedro de los Milagros. Black = Detection, Grey = No detection.
