# Supplementary material for: Integrating niche and occupancy models to infer the distribution of an endemic fossorial snake (Atractus lasallei)
Source: PLoS One. 2024 Aug 20;19(8):e0308931. doi: 10.1371/journal.pone.0308931 (PMC11335104; doi:10.1371/journal.pone.0308931)
Supplement: S4 Fig — (DOCX) [file pone.0308931.s009.docx]

**S8: Elevation distribution of *A. lasallei* detections.**


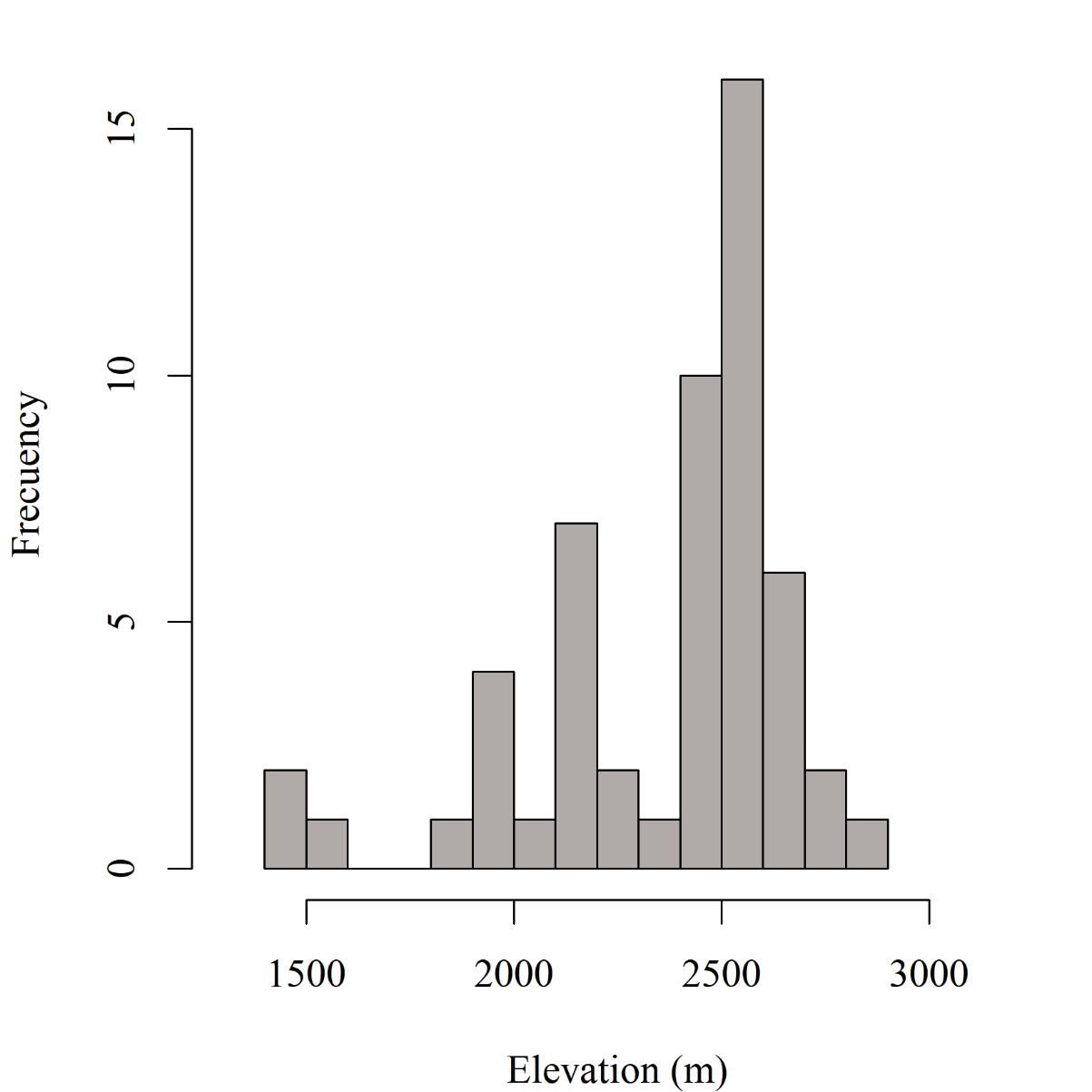


**Fig. S7.** Elevation (meters above sea level) histogram of the 54 records of *A. lasallei* used in niche modeling.
