# Supplementary material for: Integrating niche and occupancy models to infer the distribution of an endemic fossorial snake (Atractus lasallei)
Source: PLoS One. 2024 Aug 20;19(8):e0308931. doi: 10.1371/journal.pone.0308931 (PMC11335104; doi:10.1371/journal.pone.0308931)
Supplement: S5 Fig — (DOCX) [file pone.0308931.s010.docx]

**S10: Relationship between vegetation height in meters and the number of cover objects.**


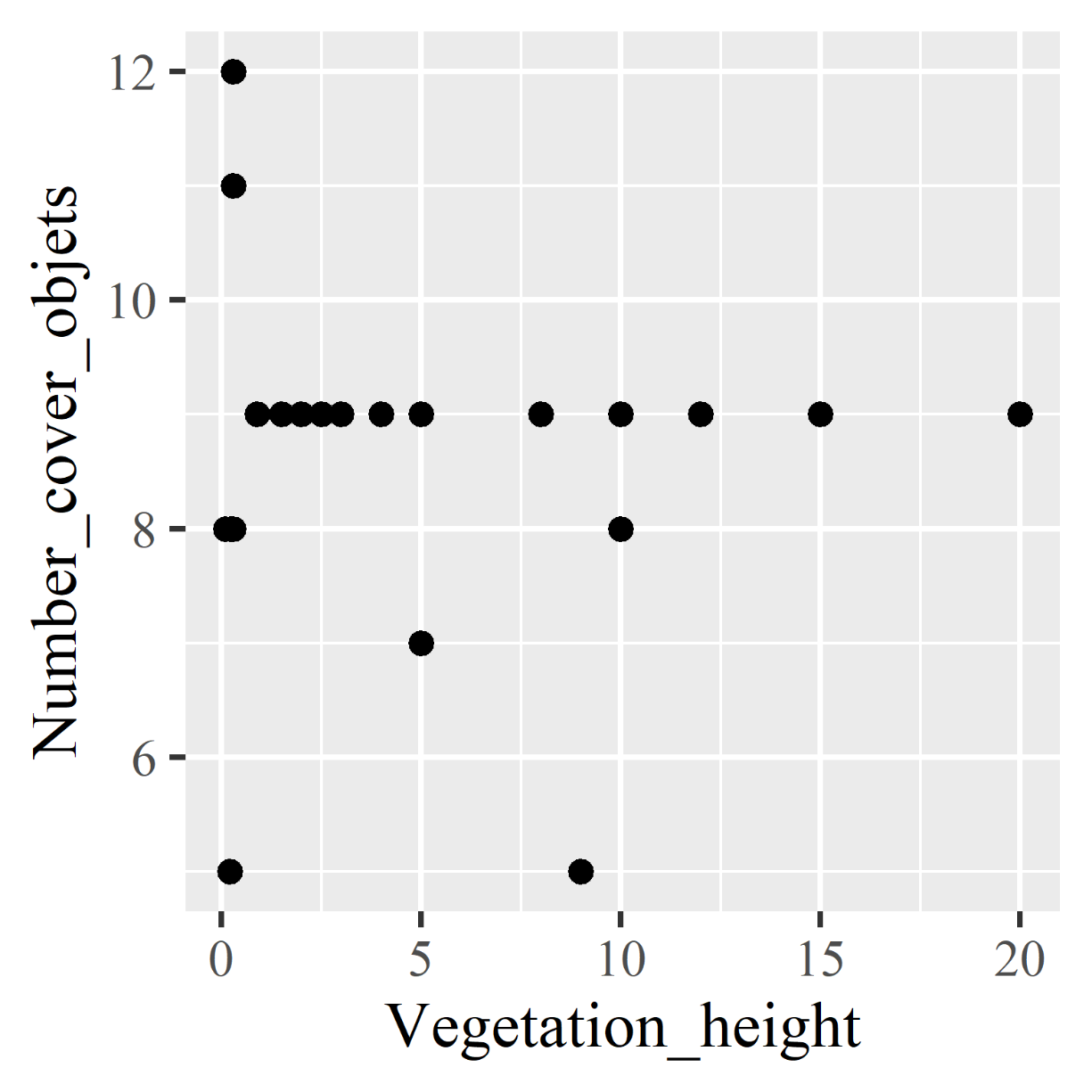


**Fig. S8.** Scatterplot of vegetation height and number of cover objects, obtained from 30 linear transects used for occupancy modelling.
