# Supplementary material for: Integrating niche and occupancy models to infer the distribution of an endemic fossorial snake (Atractus lasallei)
Source: PLoS One. 2024 Aug 20;19(8):e0308931. doi: 10.1371/journal.pone.0308931 (PMC11335104; doi:10.1371/journal.pone.0308931)
Supplement: Attachment — Submitted filename: Response to Reviewers.docx [file pone.0308931.s011.docx]

**Response to Reviewers**

**Journal requirements:**

Dear Mark A. Davis, Ph.D.
Academic Editor
PLOS ONE

Thank you for your constructive feedback on the paper, "Integrating niche and occupancy models to infer the distribution of an endemic fossorial snake (*Atractus lasallei*)". In order to meet the journal requirements:

1) We revised again the manuscript to ensure it meets PLOS ONE's style requirements, including those for file naming.
2) We included a full ethics statement in the ‘Methods’ section of the manuscript file. This section includes the full name of the ethics committee who approved our sampling protocol.
3) Regarding the figure containing a copyrighted image (Figure S3), the photo belongs to Juan David Martinez Martinez, who confirmed that he has the copyright to the image and grants us permission for its publication under a Creative Commons Attribution 4.0 International (CC BY 4.0) license. We attach a written permission from the copyright holder to publish these figures specifically under the CC BY 4.0 license.
4) We reviewed again our reference list to ensure that it is complete and correct. There are not cited papers that have been retracted.

We extend our gratitude to all Reviewers and have attended all their comments. We modified the manuscript following their suggestions. We paid special attention to the connection between the introduction and discussion, as recommended. We believe the quality of the MS has greatly improved following these changes. Please find below a detailed answer to each comment in blue.

**Review Comments to the Author**

Reviewer #1: This study adds to the literature by integrating both ecological niche and occupancy modelling to determine the large- and small-scale habitat associations of *Atractus lasallei*. The statistics and evaluation of the species distribution models were well done and well described in the results and discussion section. Additionally, the authors were very clear about how this research could be used in a conservation framework by comparing their results to the IUCN geographic distribution methods. Two of my major concerns are 1) a few places where additional information would make the manuscript clearer and 2) a lack of data following the PLOS data policy. These concerns are further outlined below. However, with the following edits, this manuscript will be a great contribution to the field.

R/ Thank you for your encouraging comments. We included additional information to improve the quality of the manuscript and made all data available through the DRYAD website. The following link can be used to download the data: (https://doi.org/10.5061/dryad.5qfttdzff).

Major comments:
1. Introduction:
a. While the discussion mentions the hierarchy of environmental filters and the association with the Grinnellian and Eltonian niches, the Introduction does not. I feel the addition of this information could help describe why you would integrate both ecological niche and occupancy modelling.

R/ All reviewers coincided on this comment. We reorganized and extended the Introduction to include all these aspects. For example, the last paragraph of the Introduction now includes (Lines 97 – 103): “*While niche models can make use of all records available but compromising temporal and spatial resolution, occupancy models require data collection under a strict sampling design that often limits the temporal and spatial extent. We take advantage of these trade-offs and predict that niche models will identify environmental requirements of the species at broad scales (e.g., Grinnelian niche), and occupancy models will identify variables more representative of essential interactions for the species (e.g., Eltonian niche). Given its fossorial-cryptic habits, we expect ground-level variables to be more informative for modelling its potential distribution, and a strong association of with habitats characterized by low vegetation such as grasslands and shrubs.*”

b. Authors mention their hypothesis in the Discussion (Lines 277-278) but do not mention them at all in the introduction – I believe authors could include hypotheses of both types of modelling in the last paragraph of the introduction.

R/ Thank you, we have now included a general hypothesis and various predictions related to both types of modelling in the last paragraph (Lines 91 – 105) of the introduction.

2. Methods:
a. For the Ecological Niche Model and Potential Distribution section, authors should add the dates at which they acquired the information. For example, “… Global Biodiversity Information Facility (accessed XXX),”

R/ Date (22 March 2022) was included in text (Lines 109–110) and data download citation was included in the references.

b. In Lines 122-123, authors mention that a Spearman correlation test was conducted to select non-correlated variables which is correct. However, in Lines 293-295 authors mention that the ground-level and atmospheric maximum temperatures were highly correlated. Due to their high correlation, they should not have been included in the same models but it is unclear in the Methods as to whether one of them was not included.

R/ We regret the confusion. As specified in lines 141-43, two sets of variables were created, one included above-ground temperature variables (set 1), and the other included atmospheric-level temperature variables (set 2). The kuenm package uses each set of variables independently in the modelling process. To clarify this, we added a sentence to ensure that is clear for readers (Lines 144-145): “*Models were calibrated with each data set independently, ensuring all variables used were not correlated.*”

c. Additionally, on the atmospheric-level variable and the variable estimated at five centimeters, there is no explanation of how the ground-level temperatures were estimated.

R/ We did not measure these variables directly, but used surfaces generated in the following study : Lembrechts JJ, van den Hoogen J, Aalto J, Ashcroft MB, De Frenne P, *et al.* Global maps of soil temperature. Global Change Biol. 2021;28:3110–3144.

We added the following sentence (Lines 142): “*Finally, two sets of variables were created, one that included two ground-level temperature variables estimated at five centimeters above the ground (S1) [41], and the second included the same two variables but measured at atmospheric level [49].”*

d. For the ecological niche models, authors mention the evaluation of “different sets of environmental variables and various model parameterizations” but do not mention what model parameterizations were tested.

R/ To clarify this we modified the paragraph as follows (lines 147-149): “*This methodology allows the evaluation of different sets of environmental variables (set 1 and set 2, S1) and various model parameterizations to ultimately identify the best model according to a set of criteria. We allowed the regularization parameter to vary from 0.1 (very strict in relation to observed values) to 5 (more flexible in relation to observed values), where 1 is the default value. We also evaluated across linear (l), quadratic (q), and linear-quadratic (lq) responses.*”

In addition, the script used (A.lasallei.Rmd) was made available (DRYAD repository) to ensure the replicability of the study (https://doi.org/10.5061/dryad.5qfttdzff).

3. Results:
a. While data are provided in S5 about where occurrence records are from (e.g., iNaturalist), to meet the criteria of the PLOS Data policy, Latitude and Longitude should also be included. Additionally, for the PLOS data policy, there should be sufficient data to replicate the study; therefore, shapefiles for the variables in the ecological niche models and data for the occupancy models should also be available.

R/ Done. All data is available at the DRYAD website: https://doi.org/10.5061/dryad.5qfttdzff.

b. For Table 1. Niche Model Results, the model names are unclear, for example, what is the consensus model, what do the numbers and lq mean?

R/ The consensus model was created using the median of the selected models (table 1). These models include atmospheric level temperature variables (Set 2). To improve the interpretation of table 1, we now expanded its description (Lines 238-242): “*Selected models that met evaluation criteria: AUC ratio, P value of partial ROC, omission rate and AICc. These models were used to create the consensus model. Model names follow this structure separated by underscores: Model (M), Regularization multiplier (0.5-5), Feature classes (F_l, F_q, and F_lq), and set of variables used (set_1 or set_2).”*

c. Table 2 and 3: All tables and figures should be able to stand alone without the paper attached therefore, all of the names used in the model names need to be defined in the table description.

R/ Done.

4. Discussion: The content of the discussion is good but the structure of a discussion should go in the order of:
a. Mini synopsis
b. Restate main findings
c. Significance and meaning (contextualization)
d. Compare results
e. Impact and applications of research
f. Currently, the discussion does not follow this structure with some of the most important impacts and applications (comparison to the IUCN and potential changes to its listing) in the middle and some of the significance and meaning/ comparing results (Lines 327-342) at the very end.

R/ Thank you. The discussion was restructured to follow – in general – the proposed order. We have eight paragraphs: the first is a mini synopsis of all results, 2-3 restate main findings about the important variables, 4-7 are a mixture of contextualization, significance and comparison to other studies, and the last states the impacts and applications of research.

Minor comments:
1. Line 28: Change “centres” to “centers” for American English, check throughout.

R/ Done.

2. Line 31: Add a general/overall conclusion sentence.

R/ We added the following sentence at the end of the abstract (Lines 31-35): “*We recommend approaches that focus on variability at different spatio-temporal scales to better comprehend the variables that affect species’ ranges and identify threats to vulnerable species. Prompt actions are needed to protect herbaceous and shrub vegetation in this region, highly demanded for agriculture and cattle grazing.*”

3. Line 41: “is” should be “are” because authors are referencing more than one thing.

R/ Done.

4. Line 50: I do not believe “conspicuous” is the correct word here; maybe “apparent” instead.

R/ Done.

5. Lines 55-56: Cite this and add to references.

R/ Done.

6. Lines 58-60: Niche modelling is more about the relationship between species occurrence data and environmental data – I am not sure what you mean by “geographic”

R/ We refer to Hutchinson's duality, The relation between environmental space – where the niche is defined – , and physical space, where the occurrence data is registered and where the geographic range is defined. We modified the sentence to (Lines 56 - 59): “*Niche modelling can be used to predict potential distribution based on the relationship between geographic (where occurrences are registered) and environmental space (where the niche is defined) [17], typically using abiotic environmental predictors (climate) to estimate suitability [18].”*

7. Line 69: Missing the word scale after fine.

R/ Done.

8. Lines 74-91: This paragraph could be structured differently to be clearer as to why these models are important for this snake in particular. Personally, I would start with the sentences in Lines 80-91 which would show the need for applying these models and then present niche and occupancy models as a viable option to learn more about its habitat use.

R/ Thank you. We followed your recommendation and modified the 4^th^ paragraph (Lines 77-90) accordingly.

9. Lines 112-115: Figure panel reference should go at the beginning of the sentence e.g., “A) Study area encompassing northern…”. Additionally, what do the green-red colors represent? That should be included in the Figure caption and a scale/legend should be included in the Figure.

R/ Description of figure was modified according to these recommendations.

10. Line 113: Capitalize “Light”

R/ Done.

11. Line 122: What raster package was used? Further, at the first mention of a program, the version should be included.

R/ The R package used was “raster” v.3.5. Text was corrected for clarity and the versions of all packages and software were added.

12. Lines 122-123: What package was used to complete the Spearman correlation test.

R/ The R package “corrplot” v.0.92 was used, it was included in text.

13. Lines 128 and 168: R package names are typically in quotes “kuenm” package. Make sure to check this throughout.

R/ Done.

14. Lines 132-133: Acronyms need to be spelled out on first use, e.g., “partial receiver operating characteristic (ROC) curve”. Check for this throughout.

R/ Done.

15. Lines 144-145: Spell out numbers below 10 (except for times and measurements). Check the rest of the document.

R/ Done.

16. Line 162: What version of QGIS?

R/ Version 3.10 was used. We included this in the text.

17. Lines 176-177: What package did you use to perform the chi-square test?

R/ We used the parboot function from the R package “unmarked” v.1.4.1. This was included in text.

18. Lines 184 and 187: Consistent with other measurements, put a space between 4 and m. Check throughout for consistency.

R/ Done.

19. Line 189-192: Use the same format as Fig 1, with (A) and (B) rather than top and bottom. Make sure to add A and B to the figure itself.

R/ Done.

20. Line 202: Add ecological before niche to stay consistent.

R/ Done.

21. Lines 224-226: See above notes for Fig 1 and Fig 2.

R/ Done.

22. Lines 233-235: Starting at “supporting” this statement is more for the discussion not the results. R/ Done.

23. Line 250: Change Atractus lasallei to A. lasallei.

R/ Done.

24. Lines 255-256: Can you reword to avoid using “with” three times?

R/ Done.

25. Line 257: Change “may provide” to “provided”.

R/Done.

26. Line 261: You should not start a sentence with an acronym so even though you already defined EOO, it should still be spelled out at the beginning of the sentence.

R/ Done.

27. Lines 276: Change “even at higher threat category” to “at an even higher threat category”.

R/ Done.

28. Line 297: What is NDVI?

R/ The term was omitted.

29. Lines 308-321: This is a great paragraph!

R/ Thanks!

30. Lines 322-326: I think this paragraph could be included with the previous paragraph since it is only two sentences.

R/ Done.

31. Lines 327-342: I think this is a great paragraph and some of this information could be added to the introduction.

R/ Done.

Reviewer #2: The manuscript “integrating niche and occupancy models to infer the distribution of an endemic fossorial snake (*Atractus lasallei*) is a well written and supported research article that meets PlosOne’s publishing criteria. The approach integrating scale into distribution through multiple methods is well done and makes for a compelling read, providing an informed understanding of distribution and occupancy. This paper provides evidence for conservation needs of *A. lasallei* and frames the conservation in the broader context of IUCN listing criteria and spatial scale. I have provided minor comments to improve on the manuscript, identifying areas for clarification and framing.

General comments:
In several sections “habitat preference” or “prefers” is used; I recommend changing this term to “habitat selection” and “selects” respectively based on Johnson (1980) terminology, where preference requires that resources are equally available.

Johnson, D. H. 1980. The comparison of usage and availability measurements for evaluating resource preference. Ecology 6: 65 – 71

R/ Thank you for pointing this out. We agree that measuring preference requires an experimental approach. We have followed your suggestion and changed the text accordingly.

The discussion brings up different niche concepts (Grinnellian and Eltonian) and extent of occurrence and area of occupancy. These concepts could be mentioned in the introduction to set up the study to strengthen the manuscript.

R/ We added them briefly in the Introduction, mostly because we did not directly measure any interactions. We included the following sentence (Lines 97-100): “*We take advantage of these trade-offs and predict that niche models will identify environmental requirements of the species at broad scales (e.g., Grinnelian niche), and occupancy models will identify variables more representative of essential interactions for the species (e.g., Eltonian niche).*”

GBIF and iNaturalist were both used for occurrence data, but GBIF pulls from iNaturalist; authors could clarify if iNaturalist occurrences were all drawn from GBIF, or if their criteria for iNaturalist resulted in additional occurrences. I appreciate the authors including the GBIF DOI to ensure reproducibility and credit.

R/ Thank you. We did initially pull the data from GBIF, but we discarded the data from iNaturalist and extracted the data from them directly, since we needed to verify the pictures associated to each record. We modified the text to provide clarity. It now reads Lines 108-120): “*Presence data were acquired from three sources: 1) specimens from biological collections, obtained from the Global Biodiversity Information Facility (accessed 22 March 2022) [35] and most of them revised in situ in the following collections: MHUA-Museo de Herpetología Universidad de Antioquia (Curator: J.M. Daza-Rojas), CSJ-h-Museo de Ciencias Naturales de La Salle (D.Z. Urrego), CBUCES-D-Colecciones Biológicas Universidad CES (J.C. Duque); 2) iNaturalist records obtained directly from them (accessed March-May 2022; we did not use iNaturalist records from GBIF) by searching Atractus records in the northwest of Colombia that included pictures that allowed verification through morphology (coloration patterns, and scale counts when possible), and 3) individuals encountered during the field phase for occupancy models. Identification of individuals was based on the original species description and taxonomic revisions of the genus [28, 33]. Further, a geographical filter was applied to presence records that were within 1 km of each other to reduce spatial autocorrelation [36, 37].”.*

The GBIF DOI was included in the references.

For the data used in the distribution models, please include years of the occurrence records. Given that some predictors are dynamic (precipitation, temperature), providing a temporal range of the occurrence data used will inform interpretation of the model results. I do appreciate the mention of only using abiotic variables used in the ENM due temporal effects (Line 341-342), but more information could be provided to validate that those abiotic factors are either constant or likely representative during the time the occurrence was observed.

R/ The year of the occurrence record was included in Supplementary table (S5). The climatic variables used refer to long-term (>10 years) averages. In our specific case, we used the CHELSA dataset that includes averages from 1980-2010. These variables represent long term climate spatial and seasonal variability. Variables used in occupancy analyses (e.g., ground temperature and soil moisture) on the other hand represent observations taken during sampling.

The parameter selection based on ecologically relevant variables is well done (Lines 117-120), and the authors have selected variables relevant to their study species.

For the occupancy models (lines 152-177), I recommend introducing the approach of iteratively running models to determine detectability prior to determining occupancy before discussing the parameters included in the models. On first read, I was concerned that the same parameter was used for detection and occupancy. I will note that the methods are appropriate and well done, so this is just a paragraph organization comment. Leading with the structured analysis approach before mentioning the covariates would strengthen this section.

R/ Thank you. We rephrased this paragraph (Lines 176-195) and introduced the approach with the following sentence: “*To identify the best models, we first established the best detection model assuming constant occupancy, and then we used this detection model in all occupancy models [54].*”.

PlosOne submission guidelines do not have information on keywords; if keywords are allowed, some of the keywords like “Conservation” or “Ecology” seem incredibly broad; authors should consider if there are words that would be better. Methodology terms may be an option to consider such as “maxent” or “single-season occupancy model” so people looking for those frameworks will find the article.

R/ Thank you. We deleted this section since – as you correctly pointed out – PloS ONE does not include a section for keywords.

Line comments:

Line 24: I recommend:

“We modeled the potential distribution of A. lasallei based on ecological niche theory [using maxent], and habitat use was characterized while accounting for imperfect detection using a [single-season] occupancy model.”

R/ Done.

Line 25: “that” should not be in italics.

R/ Done.

Line 55-56: The statement that models that account for detection through multiple site visits is more recent than broad scale species distribution models needs a citation, or at least needs more context. Both methods gained strong applications in the 2000s with MacKenzie (2002) introducing methodology for detection probabilities <1 and Phillips et al. (2006) introducing species distribution modeling through a maximum entropy approach. I think reframing this to be how the approaches are complimentary accounting for different spatial scales of distribution and site selection would strengthen the support for your methods and results.

R/ Thank you. The introduction was reorganized and rewritten to improve clarity. We also included the citations of the first niche modeling algorithm (BIOCLIM; Busby 1991) to better support our claim that occupancy models appeared more recently. Throughout the introduction though, we emphasize how the integration of both approaches should improve our understanding about species’ geographic ranges.

Line 74: I recommend changing “viable” to “complimentary”.

R/ Done.

Line 106: 1 km is a reasonable spatial filtering distance; is there a citation that could be included on A. lasallei dispersal distance that would further support this spatial filtering and provide an ecological context for selecting 1 km? If not, 1 km as stated is sufficient.

R/ In general, the information related to the dispersal capacity of snakes is poor. Particularly for the genus *Atractus* there are no ecological studies on this subject, partly due to their semi-fossorial habits. Therefore, we assumed 1 km as a reasonable distance for fossorial-cryptic snakes.

Line 132: for the partial ROC analysis, if done in the same kuenm R package, please include a citation to Peterson et al. (2008) to clarify methodology.

R/ Done.

Peterson, A. T., Papeş, M., & Soberón, J. (2008). Rethinking receiver operating characteristic analysis applications in ecological niche modeling. Ecological Modelling, 213, 63–72. 

Line 159: in-line citation is incorrectly formatted and has “(Lembrechts et al., 2021)[40]).” Please remove “(Lembrechts et al., 2021)” and just keep the numeric reference.

R/ Done.

Line 219: clarify where the threshold 0.096 came from.

R/ Done. It now reads (Lines244-246): “*The predicted potential distribution area for the species covers 6182 km² after applying the 0.096 threshold, obtained from averaging the 10 percentile training presence thresholds from the best models (Table 1), to generate a presence/absence map.*”

Line 286: change to “resulting in the death of [snakes].” as there could be ambiguity if the human-snake conflict results in the death of people or snakes.

R/ Done.

Line 289: “The species prefers sites with low slopes (< 5 degrees);” in the results (Line 209-2010), it’s stated that the species prefer slopes below 20 degrees. From the ENM response curves (S6), it appears that there is selection for low slopes with slopes of 0 degrees being optimal. Check these results and clarify for consistency.

R/ The niche model results indicated the species is associated with flat terrains (<10°) despite this not being the most common characteristic within the accessible area. However, there are observations in terrain with up to 20° slopes. To avoid confusion, we opted to use <10° slope to describe the species selection throughout the text.

Line 309: change “canopy height” to “vegetation height” for consistency.

R/ Done.

Tables and Figures

Table 1: spell out the model or identify what parameters are in the model as model names “M_0.5_F_lq_set_2” are not informative to a reader. Write out and define Pval and pROC in the caption.

R/ Thank you. Table 1 legend now reads (line 238-2442): “*Selected models that met evaluation criteria: AUC ratio, probability value (Pval) of partial ROC (pROC), omission rate and AICc. These models were used to create the consensus model. Model names follow this structure separated by underscores: Model (M), Regularization multiplier (0.5-5), Feature classes (F_l, F_q, and F_lq), and set of variables used (set_1 or set_2).”*

Reviewer #3: General Comments:
This manuscript evaluates the distribution of a cryptic snake, *Atractus lasallei*, using two complementary techniques, the Maxent algorithm and occupancy modeling. The authors use the former to evaluate the potential distribution (ecological niche) of *A. lasallei* based on presence-only records and relatively large spatial scale predictors, and the latter for targeted surveys of transects to identify fine-scale associations of *A. lasallei* occurrence while correcting for imperfect detection. The authors identified slope, annual precipitation, evapotranspiration, and maximum temperature as important variables defining the ecological niche of *A. lasallei*, whereas occurrence at specific sites within these large-scale constraints was associated with lower vegetation height (e.g., grasslands and shrublands). The authors also identified that the availability of cover objects was positively associated with detection probability in the occupancy models. The authors compare their results to the IUCN concepts of Extent of Occurrence (EOO) and Area of Occupancy (AOO) and suggest that *A. lasallei* could be considered Vulnerable using these criteria. The authors also compare the use of presence-only species distribution models to the Grinnellian (or potential) niche, and occupancy to the Eltonian (or realized) niche. Finally, the authors indicate how occurrence of the snake in flat areas with low vegetation heights increases the likelihood of negative interactions with humans that often result in the death of the snake.

I found the manuscript generally well-written. The Introduction provided good background information on the importance of modeling distributions of species and the strengths and weaknesses of the different approaches used in the manuscript. The Methods were appropriate, and the supplemental information supported the Methods and Results well. The authors presented the Results clearly, and the Discussion nicely put the study into context without straying too far or becoming too speculative.

I have relatively few concerns with the manuscript, and most of these appear in the Specific Comments below. In addition to those comments, there are a few items that, in my opinion, warrant some further consideration:
• The authors suggest that the primary difference between presence-only species distribution models and occupancy models is spatial scale, but in my mind that is not the case. In theory, both methods could be applied at the same spatial scale, it is just that occupancy modeling requires accounting for the detection process, which is a cost in terms of study design and field sampling, but a huge benefit in terms of accounting for sampling bias and providing reliable inference. Therefore, the separation of these two methods by spatial scale is somewhat artificial on theoretical grounds, but does have some merit in terms of implementation in the field. I would suggest that occupancy modeling be used in preference to presence-only species distribution modeling as a general guide, but I understand that this is generally not possible over the range of most species.

R/ First, we would like to clarify that we agree with the reviewer that the primary difference between the two modeling approaches is not the scale at which they are applied, but rather the recognition of imperfect detection and the sampling scheme (sites are revisited in occupancy models). We modified the manuscript introduction and discussion to avoid this interpretation. We agree that both methods could be applied at the same spatial scale. However, we do take advantage that, in practice, mostly due to logistical restrictions, occupancy models are implemented at relatively smaller spatial and temporal extents, using variables measured at finer resolutions than in niche modelling. We believe these variables could be more indicative of the spatial and temporal variation of interactions, but we don´t have any type of empirical support for that.

• The comparison of the two methods to Grinnellian and Eltonian niches is interesting, but here, too, I think the primary difference is more than spatial scale. As I interpret them, the Grinnellian niche is the areas the species could potentially occupy if all biotic constraints were absent (i.e., plenty of resources, competition and predation were not limiting factors, etc.) and the Eltonian niche is where the species occurs after accounting for these biotic interactions. So in my mind, these aren’t necessarily about spatial scale (limiting abiotic factors could change over very small spatial scales for some species), but rather about the variables included in the model (either presence-only SDM or occupancy). In this sense, I wonder if vegetation height is, for these snakes, part of the potential or realized niche. The biotic interactions of the snakes with the vegetation are very limited (i.e., they do not eat the vegetation, it doesn’t compete with them, and it does not eat them), but the vegetation could have a very large effect on the physical environment (temperature, humidity, structural attributes, etc.) that influence the snakes directly and indirectly via their prey and predators (including humans). I suggest either providing more nuance to these concepts and separating them from spatial scale and the type of model used or removing this section of the Discussion.

R/ Thanks again for your valuable comments. We agree that vegetation height in itself does not directly represent an interaction. It would be fantastic to have quantified prey availability, or abundance of potential competitors, or parasite prevalence, but usually, these variables were out of our reach. Coarse-grained climatic variables may be a better at representing the fundamental niche, but often, in geography, the species is not present in areas with suitable climate due to negative or lack of positive interactions. The hypothesis (Eltonian noise hypothesis) is that the spatial variability of the latter variables expresses at a finer grain (like the one approximated by vegetation height). In conclusion, we do recognize that we did not measure interactions directly, but we assume that vegetation height can be used as a proxy for some interactions. For example, based on our observations, these snakes are very selective of earthworms as prey, which are very abundant in places with low vegetation and scarce in forest.

• Similar arguments apply to the authors’ comparison of their results to the IUCN concepts of EOO and AOO. Here, too, either modeling framework could inform these metrics, though I agree that occupancy modeling is much better-suited to AOO because of its ability to correct estimates of occurrence for sampling bias.

R/ In this sense, both EOOs and AOOs are defined according to the method proposed by the IUCN, which is why we interpret our results as actual area of distribution and not as AOOs. In this case we propose that the integration of different distribution modeling methods generates more accurate distribution estimates than those obtained from a single methodology, and thus better informs about the conservation status of the species.

In addition to these general comments, I reference specific comments by line number below.


Specific Comments:
Line 25: Remove italics from “that.”

R/ Done.

Line 26: Replace “exhibits preferences for” with “selects.”

R/ To avoid conceptual confusion related to habitat related concepts, we decide to change habitat preferences for associations throughout all text.

Hall, L. S., Krausman, P. R. & Morrison, M. L. (1997). The habitat concept and a plea for standard terminology. Wildlife Society Bulletin, 25(1), 173–182.

Line 48: Delete “the.”

R/ Done.

Line 64: I recommend replacing “presence-absence” with “presence-nondetection” to highlight that absence is difficult to ascertain, especially with cryptic snakes.

R/ Done.

Line 67: Replace “it’s” with “it is.”

R/ Done.

Line 69: Insert “scale” after “fine.”

R/ Done.

Lines 70–71: Occupancy models do not require that populations be closed (i.e., no change in abundance because of birth, death, immigration, or emigration), but single-season occupancy models do assume that sites are closed to changes in occupancy status (i.e., empty sites are not colonized and occupied sites do not become extirpated within the sampling period). The other assumptions can be, and in fact often are, relaxed. Applying covariates to occupancy and detection implies that these are not constant, but rather vary with environmental or survey conditions. I suggest rewording this sentence.

R/ Text was modified to clarify modelling assumptions. Lines 70-74: “*Researchers must revisit a series of sites and acquire information during a specific time and spatial intervals. Sampling design must fulfil a series of assumptions (that can sometimes be relaxed), such as the closure assumption (sites are closed to changes in occupancy status within sampling period) and independent detection histories.*”

Line 88: Delete “up.”

R/ Done.

Line 114: Capitalize “light” at the beginning of the sentence.

R/ Done.

Line 172: This is a large number of models, especially for 30 transects. Given the exploratory (and predictive, in the sense that the selective model is used for mapping rather than causal inference) nature of the work, however, I think this is OK. It might be worth noting that 20 of the models were for the detection component with constant occupancy, and the remaining models were for the occupancy component.

R/ We included this information in the paragraph (Lines 197-198).

Line 184: Replace the comma with a semicolon.

R/ Done.

Lines 185–187: Yes, but were vegetation height and number of cover objects related at all? The detection probabilities are very low with fewer than 10 cover objects, resulting in a lot of uncertainty in the relationship between vegetation height and occupancy. Given this level of uncertainty, 4 m vegetation height seems like it could exclude a reasonably large proportion of area suitable for the snakes. It would be good to present some indication of the distribution of the number of cover objects and vegetation height among transects either by placing tick marks along the x-axes in Fig. 2 or as histograms (or better, a scatterplot of vegetation height vs. number of cover objects) in a supplemental figure.

R/ Done. We include the figure in supplementary material.

Lines 206–207: This belongs in the discussion.

R/ Done.

Line 209: Replace “has preference for” with “is associated with.” Preference is generally restricted to mean that given equal availability of resources (usually in a laboratory setting), an animal would choose resource X over resource Y.

R/ Done.

Lines 209–212: Some of these response curves have distinct peaks. It might be desirable to narrow the reported range of values associated with snake detection. For example, flatter slopes (< 10°), precipitation between 2000 and 3500 mm, evapotranspiration between 80 and 120, and maximum temperature between 17 and 22 (lower values have a lot of uncertainty) seem to me to be particularly high in the response curves.

R/ Text was changed following your suggestions. Extreme values have higher uncertainty but given our experience, we trust than optimum values were well identified by reported response curves.

Line 218: Remove period from “6182 km2” to avoid confusing readers in some countries.

R/ Done.

Line 251: Delete comma following “climatic” and replace “were” with “was.”

R/ Done.

Line 253: Replace “prefers” with “is associated with.”

R/ Done.

Line 254: Replace “terrains” with “terrain.”

R/ Done.

Line 255: Replace “a preference for” with “an association with.”

R/ Done.

Line 263: Delete “and” where it precedes “usually.”

R/ Done.

Line 265: Delete comma.

R/ Done.

Line 276: Replace “even at” with “at an even.”

R/ Done.

Line 284: Replace “preys” with “prey.”

R/ Done.

Line 289: Replace “prefers” with “is associated with” and delete comma.

R/ Done.

Lines 290–297: Ground temperature is a calculated expectation, and I would expect that this would vary substantially on a much smaller than 1 km2 spatial scale that is more relevant to the snakes (in addition to the mechanisms described here). Perhaps that is another reason why it was not particularly predictive. I agree with the sentence on lines 297–298; such microclimatic data are difficult to obtain across larger spatial scales, though.

R/ Thank you. This observation was incorporated in the discussion, Lines 306-308: “*Ground level variables should be more informative at finer spatial and temporal scales, which unfortunately are not currently available for the area of interest. We suggest exploring alternative sources of microclimatic data [62, 63].*”

Lines 315–318: Agreed.

R/ Thanks.

Line 321: Replace “the detection histories” with “detection probabilities.”

R/ Done.

Lines 322–326: The recommendation to use a removal design is particularly efficient when species are common, as for A. lasallei in grasslands. Specht et al. (2017) provide particularly useful guidance on alternative occupancy designs under different conditions of ψ and p.

R/ Thanks, we rewrote the sentence and mentioned the paper.

Line 336: Replace “preys” with “prey.”

R/ Done.

Fig. 2. I recommend replacing the word “Quantity” in the x-axis label of the top panel with “Number.”

R/ Done.


Literature Cited:
Specht, H. M., H. T. Reich, F. Iannarilli, M. R. Edwards, S. P. Stapleton, M. D. Weegman, M. K. Johnson, B. J. Yohannes, and T. W. Arnold. 2017. Occupancy surveys with conditional replicates: An alternative sampling design for rare species. Methods in Ecology and Evolution 8:1725–1734.
